# Learning From a Massive Open Online COVID-19 Vaccination Training Experience: Survey Study

**DOI:** 10.2196/33455

**Published:** 2021-12-03

**Authors:** Shoshanna Goldin, So Yeon Joyce Kong, Anna Tokar, Heini Utunen, Ngouille Ndiaye, Jhilmil Bahl, Ranil Appuhamy, Ann Moen

**Affiliations:** 1 Influenza Preparedness and Response Organisation Mondiale de la Santé Genève Switzerland; 2 Strategic Research Laerdal Medical Stavanger Norway; 3 Learning and Capacity Development Unit WHO Health Emergencies Program Organisation Mondiale de la Santé Genève Switzerland; 4 Department of Immunization, Vaccines and Biologicals Organisation Mondiale de la Santé Genève Switzerland

**Keywords:** COVID-19, vaccination, training, massive open online course, pandemic, vaccine, education, online education, preparation, evaluation, user experience, challenge, impact, knowledge, interest

## Abstract

**Background:**

To prepare key stakeholders for the global COVID-19 vaccination rollout, the World Health Organization and partners developed online vaccination training packages. The online course was launched in December 2020 on the OpenWHO learning platform. This paper presents the findings of an evaluation of this course.

**Objective:**

The aim of this evaluation was to provide insights into user experiences and challenges, measure the impact of the course in terms of knowledge gained, and anticipate potential interest in future online vaccination courses.

**Methods:**

The primary source of data was the anonymized information on course participants, enrollment, completion, and scores from the OpenWHO platform’s statistical data and metric reporting system. Data from the OpenWHO platform were analyzed from the opening of the courses in mid-December 2020 to mid-April 2021. In addition, a learner feedback survey was sent by email to all course participants to complete within a 3-week period (March 19 to April 9, 2021). The survey was designed to determine the perceived strengths and weaknesses of the training packages and to understand barriers to access.

**Results:**

During the study period, 53,593 learners enrolled in the course. Of them, 30,034 (56.0%) completed the course, which is substantially higher than the industry benchmark of 5%-10% for a massive open online course (MOOC). Overall, learners averaged 76.5% on the prequiz compared to 85% on the postquiz, resulting in an increase in average score of 9%. A total of 2019 learners from the course participated in the survey. Nearly 98% (n=1647 fully agree, n=308 somewhat agree; N=1986 survey respondents excluding missing values) of respondents fully or somewhat agreed that they had more confidence in their ability to support COVID-19 vaccination following completion of this course.

**Conclusions:**

The online vaccine training was well received by the target audience, with a measurable impact on knowledge gained. The key benefits of online training were the convenience, self-paced nature, access to downloadable material, and ability to replay material, as well as an increased ability to concentrate. Online training was identified as a timely, cost-effective way of delivering essential training to a large number of people to prepare for the COVID-19 vaccination rollout.

## Introduction

To address the need for timely training on COVID-19 vaccination, the Access to COVID-19 Tools Accelerator’s Country Readiness and Delivery (CRD) workstream rapidly produced the *COVID-19 vaccination training for health workers* course. The course was launched on the OpenWHO platform in mid-December 2020. The objective was to ensure that health workers responsible for COVID-19 vaccination deployment had timely access to World Health Organization (WHO) recommendations and information that could help them prepare for a safe and efficient vaccine rollout. Although the course was targeted to health workers, it was open to all and was accessed by others, such as policy makers, community leaders, and students.

As the COVID-19 pandemic limited travel and the ability to gather learners together in a typical classroom environment, the health workers course was developed as an online learning curriculum. The health workers course includes a series of video lectures presented by technical experts, with accompanying multiple-choice questions delivered before and after the lectures. The transcripts and videos are downloadable. The health workers course contains 6 modules and provides information on organizing the vaccination session, including infection prevention and control measures; COVID-19 vaccine storage, handling, administration, and safe disposal; recording and monitoring, including adverse events following immunization (AEFI); and communication with the community.

OpenWHO is one of the WHO’s online learning platforms, offering free online courses with the aim to improve responses to health emergencies [1]. The platform hosts over 100 courses on COVID-19 and other health topics, has over 5 million enrollments, and offers courses in 55 languages. Within the OpenWHO platform, registration includes an option to self-declare the registrant's occupation. The occupation selection is not validated and may be subjective, depending on the registrant’s consideration of their role in the workforce.

Given that online learning to prepare for vaccine introduction is a relatively new approach for many countries, this course was evaluated to understand participants’ online learning experience. The aim of this analysis is to increase the effectiveness of OpenWHO training packages and to plan for future online immunization learning.

The COVID-19 pandemic emphasized the need for trusted, accurate information to help health workers and the public respond to the outbreak. Online learning to prepare for vaccine introduction is a relatively new approach [2]. This paper provides an overview of the OpenWHO COVID-19 vaccine introduction training for health workers, shares insights on participants’ online learning experiences, and provides key findings that can be used to improve future real-time online training.

The title of the course (*COVID-19 vaccination training for health workers*) uses the WHO definition of health workers as all people engaged in actions whose primary intent is to enhance health.

## Methods

The analysis is based on quantitative data collected from the OpenWHO integrated statistical data and analytics reporting system. Anonymized course reports data sets were extracted from the OpenWHO reporting tool, providing raw data including basic demographics on OpenWHO users (eg, self-declared age, gender, professional affiliation, and nationality information), which were entered at the time of registration to the platform. OpenWHO course reports also include course-specific learners’ performance and course activity indicators (such as module completion), including videos, self-assessments, download activity status, quiz performance, and obtention of the certificate, as well as tracked average session duration and time-stamped activity usage patterns.

Course registration and completion data captured by the OpenWHO analytics systems were analyzed to understand user demographics, certifications, and dropout rates. Completion of the course was defined as watching all videos and completing the postquiz with a score of at least 80%. Questions were scored as correct or incorrect—no partial credit was granted. Demographic information, including age, gender, and professional affiliation were not mandatory; therefore, analyses on these variables were based on the learners who provided information voluntarily. The course activity data of individual learners—including modules visited, videos watched, and resources downloaded—were collected and analyzed to understand the usability of the training course. Data on the scores of individual learners for pre- and postquizzes were collected and used in the analysis to measure knowledge gained. Learner’s knowledge gain was assessed by comparing average postquiz scores to prequiz scores (where data were available). Both pre- and postquizzes had the same questions. Pre- and postquizzes were included before and after each module, respectively. The number of questions was limited (2-4 questions per module) to avoid overburdening the learners. Learners had a single attempt for prequizzes, but multiple attempts were allowed for postquizzes. For each learner’s postquiz scores, the scores from the first postquiz attempts were used. Due to the limited number of questions per module, statistical significance by module could not be analyzed. Analysis of the course data was based on the total number of enrolled learners from the course opening date (December 18, 2020) until the date when the course analyses for this paper began (April 18, 2021).

In addition, an exit survey was added at the end of the course to collect participant feedback on course content to better understand the usability and virtual learning experiences of the learners, as well as strengths, weaknesses, and barriers of the training package. The learner feedback survey was composed of 21 questions (Multimedia Appendix 1). The survey was implemented on the OpenWHO platform and opened for a period of 3 weeks (March 19-April 9, 2021). For those learners who enrolled and completed the health workers course prior to March 19, 2021, survey invitation emails were sent. The survey was voluntary and indicated that it was conducted to collect feedback and that results may be used for research purposes.

The two sets of anonymized statistical data from the course report and survey data were overlaid by using a unique pseudo-ID for each OpenWHO learner, thus allowing the two data sets to be merged for analysis.

All analyses were conducted using Python (version 3.8.3; Python Software Foundation). OpenWHO data and the survey data were collected in line with the OpenWHO Terms of Use, which every enrolled user accepts. All OpenWHO users agree to the following statement, which was provided by the Office of the Legal Counsel of the WHO: “Records of your participation in OpenWHO courses may be used for education research. In the interest of this research, you may be exposed to variations in the course content. Research findings will typically be reported at the aggregate level. Your personal identity will not be publicly disclosed in any research findings without your express consent.” As the survey was conducted to provide feedback on the course, ethical clearance was not required.

## Results

### Summary Statistics

During the study period, the total number of enrolled learners was 53,595. Of all enrolled learners in the health workers course, 30,034 (56.0%) completed the course. Out of 2019 survey participants, 1857 (92.0%) completed the course and 432 (8.0%) did not. All survey responses were included in the survey analysis, as this paper considers the knowledge gained and experiences of participants who did and did not complete the course.

Demographic characteristics of the enrolled learners for the course are described in Table 1. Of the enrolled learners, 34,746 (64.8%) provided their gender, 33,557 (62.6%) provided their age, and 46,909 (87.5%) provided their professional affiliation. There were more female learners (n=18,388, 52.9%) than male learners (n=16,311, 47.0%) and the age group of 20-29 years was most dominant in the course (n=11,444, 34.1%). The top 3 professional affiliations of learners were the following: health care professionals (n=21,487, 45.8%), students (n=6874, 14.7%), and ministry of health officials (n=4666, 9.9%).

The course was also translated into 11 additional languages: Arabic, Chinese, Dutch, French, Indonesian, Kazak, Macedonian, Spanish, Russian, Portuguese, and Vietnamese. This analysis considers the English version, as it was the original course launched and it has the largest enrollment of the language versions. Learners from 191 countries participated in the English version of the course. The four countries with the highest number of participants in the English course were India (n=3998, 11.8%), Philippines (n=2700, 7.9%), Nigeria (n=2297, 6.8%), and Rwanda (n=2163, 6.4%).

**Figure 1:**
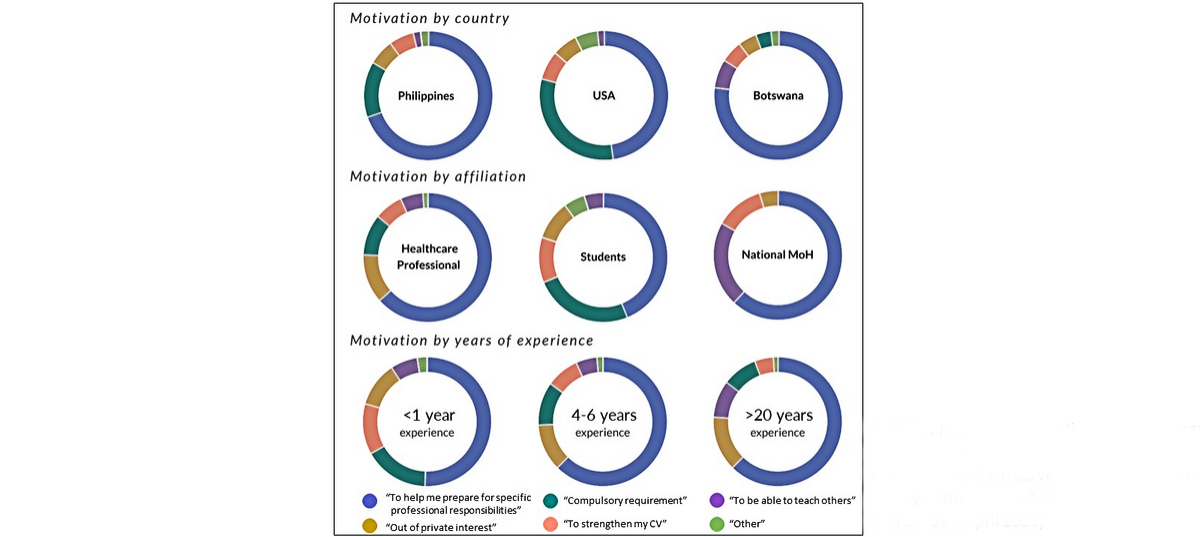
Survey participants' motivation for taking the health workers course by selected country, professional affiliation, and years of experience (total survey participants=2019; survey period from March 19 to April 9, 2021). MoH: Ministry of Health; USA: United States of America.

When asked about their motivation to enroll in the health workers course, 58.5% (n=1177) of survey respondents replied that they participated in the course to prepare themselves for specific professional responsibilities, 12.6% (n=255) out of private interest, 10.9% (n=220) because it was mandatory, 8.0% (n=161) to strengthen their resume, 7.0% (n=142) to be able to teach others, and 3.0% (n=64) for other and unknown reasons. However, there were substantial differences in motivation between countries, professional affiliations, and years of experience (Figure 1 and Table S1 in Multimedia Appendix 2 for numerical values). For example, most learners from the United States were students and took the course because it was required. In comparison, a large proportion of the learners from Ministries of Health took the course to be able to teach others.

**Table 1:** Demographic characteristics of the total enrolled learners in the English version of the *COVID-19 vaccination training for health workers* course on OpenWHO from December 18, 2020, to April 18, 2021.

Characteristics		Values, n (%)
Total enrollments		53,595 (100)
Number of learners that completed the course		30,034 (56)
**Gender^a^**		
	Female	18,388 (52.9)
	Male	16,311 (47)
	Other	47 (0.1)
**Age group (years)^a^**		
	<20	1279 (3.8)
	20-29	11,444 (34.1)
	30-39	10,756 (32)
	40-49	5711 (17)
	50-59	3146 (9.4)
	60-69	1094 (3.3)
	>70	127 (0.4)
**Professional affiliation (top 3)^a^**		
	Health care professionals	21,487 (45.8)
	Students	6874 (14.7)
	Health ministry	4666 (9.9)
**Country of residence (top 4)^a^**		
	India	3998 (11.8)
	Philippines	2700 (7.9)
	Nigeria	2297 (6.8)
	Rwanda	2163 (6.4)

^a^Among those who provided information.

### Usability of Course

**Figure 2:**
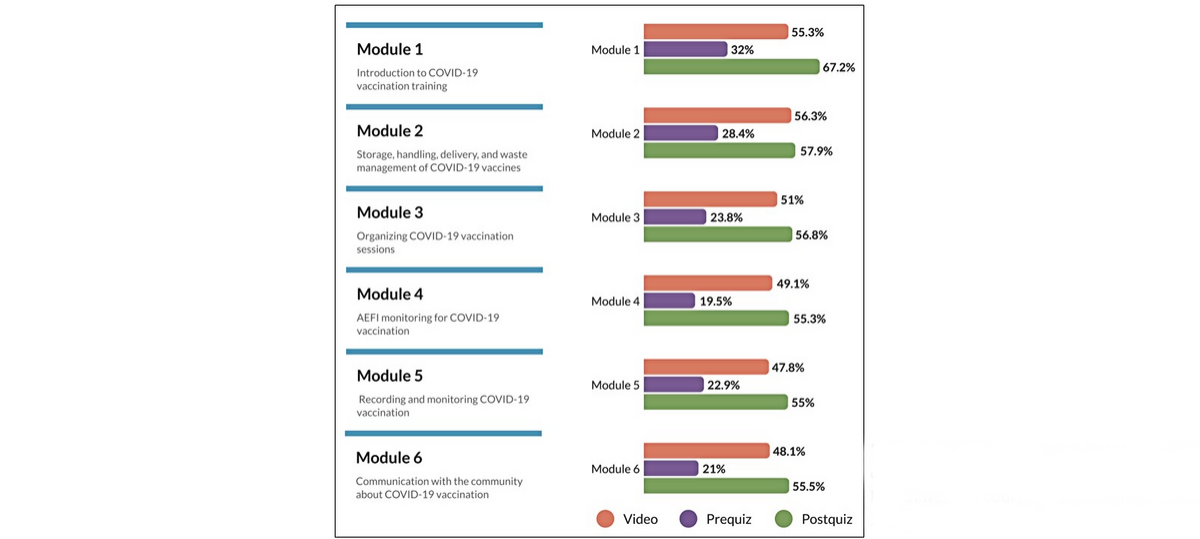
Usability (by average percent completion) of modules by total enrolled learners (n=53,595) in the COVID-19 vaccination training for health workers course on OpenWHO from December 18, 2020, to April 18, 2021. AEFI: adverse events following immunization.

Survey respondents indicated that they primarily watched the videos as the main resource for the training, which corresponds to the intended training method. Based on the total enrollment course data, on average, Module 2, which focused on supply chains and logistics, was the most watched video of the course, while Module 5, which focused on reporting and monitoring COVID-19 vaccination, was the least watched video among all enrolled learners (Figure 2). More than half of the survey respondents also read the transcripts (n=1070, 53.0%) and downloaded the presentations (n=1083, 53.6%).

For all modules, the postquiz was the course component deemed most useful by the enrolled learners. Although the quizzes were not mandatory, the postquizzes were highly used.

Males aged 40-49 years and females aged 50- 59 years were most likely to complete the course. The demographics of learners least likely to complete the course were females under the age of 20 years and learners from a health expert group or other ministry.

For this course, learners spent a median of 25.7 minutes per session and typically completed the course in three sessions (total duration of 72.1 minutes). As the run time for all videos in this course is approximately 1.5 hours, learners may have played videos at a faster speed (OpenWHO allows for participants to speed up the videos by up to 2 times the speed of the original recording) or skipped some parts of the videos.

### Knowledge and Confidence Gained

As the increase in scores varied by module, the breakdown by module is included below, along with the average change in score from pre- to postquiz. Notably, the module with the highest increase in score was Module 4, which focuses on AEFI monitoring. In Module 4, learners averaged 62% on the prequiz and 78% on the postquiz, for an overall increase of 16%. Overall, learners averaged 76% on the prequiz compared to 85% on the postquiz, resulting in an increase in average score of 9% (Table 2).

Survey respondents were asked whether they had more confidence in their ability to perform their professional role related to COVID-19 vaccination after this training. Overall, nearly 83% (n=1647) of respondents fully agreed with this statement and an additional 15.5% (n=308) somewhat agreed with this statement. Among the top 3 professional affiliation groups, health care professionals were most likely to fully agree with this statement (n=1136, 85.4%) compared to students (n=171, 77.7%) and those working in the public health sector (n=66, 71.0%). Individuals with 4-6 years of experience in their field had the highest “fully agree” rate (n=240, 86.1%), while those with <1 year of experience had the lowest “fully agree” rate (n=196, 78.7%).

**Table 2:** Pre- and postquiz scores of 53,595 enrolled learners for the *COVID-19 vaccination training for health workers* course on OpenWHO from December 18, 2020, to April 18, 2021.

Modules	Prequiz score^a^, %	Postquiz score^a^, %	Improvement, %	Number of questions
Module 1: Introduction to COVID-19 vaccination training	88	93	5	2
Module 2: Storage, handling, delivery, and waste management of COVID-19 vaccines	81	88	7	2
Module 3: Organizing COVID-19 vaccination sessions	72	85	13	4
Module 4: AEFI^b^ monitoring for COVID-19 vaccination	62	78	16	3
Module 5: Recording and monitoring COVID-19 vaccination	75	84	9	2
Module 6: Communication with the community about COVID-19 vaccination	81	81	0	2
Total	76	85	9	15

^a^Among those who participated in the quiz.

^b^AEFI: adverse events following immunization.

### Considerations for Future Virtual Trainings

The health workers course was particularly well received by health care professionals. About 99% (n=1966) of survey respondents indicated that they would recommend this course to others, with 91.3% (n=1832) fully agreeing and 7.6% (n=134) somewhat agreeing. Among the top 3 professional affiliation groups, health care professionals had the highest fully agree rate (n=1244, 93.7%), while students had the lowest (n=188, 86.2%).

When asked their preferred training methods, 65.6% (n=1293) of the survey respondents preferred online training, 29.3% (n=579) preferred blended (combination of online and in-person training), 3.7% (n=73) preferred in-person training, and 1.4% (n=27) were unsure. The reasons respondents preferred online training (multiple responses were possible) included the convenience of the timing (n=1204, 59.6%), the self-paced nature (n=1039, 51.5%), the ability to download the materials (n=907, 44.9%), the ability to replay sections (n=890, 44.1%), and the increased ability to concentrate (n=520, 25.8%). As these responses were from learners who participated in the online training and completed the online survey, it is important to note that this may reflect an overestimate of overall willingness to participate in online learning among the general population.

When asked about areas for future improvement, 32.9% (n=604) of survey respondents requested that OpenWHO offer more COVID-19 vaccination courses (particularly vaccine-specific resources).

In addition, 17.9% (n=362) of survey respondents requested the course be available in their national language and 4% (n=81) asked for more interaction with technical experts.

At the time of the submission of this article (July 2021), the health workers course has been provided in 12 languages and has had more than 110,000 enrollments (Table 3). The average completion rate is 65% and the highest is 89%, for the Spanish-language course.

**Table 3:** Total number of enrollments and completion certificates awarded by language version for the *COVID-19 vaccination training for health workers* course on OpenWHO from December 18, 2020, to April 18, 2021.

Course number in order of launch date	Language	Number of enrollments	Number of certificates (completion rate)
		N=110,836	N=71,770
1	English	68,267	37,284 (55)
2	Bahasa	4660	2786 (60)
3	Russian	350	159 (45)
4	Macedonian	84	39 (46)
5	Chinese	424	240 (57)
6	Arabic	1324	665 (50)
7	Spanish	32,672	29,178 (89)
8	French	1959	970 (50)
9	Portuguese	501	209 (42)
10	Vietnamese	394	195 (49)
11	Dutch	182	44 (24)
12	Kazakh	19	1 (5)

## Discussion

### Principal Findings

Although the completion rate for this course was substantially higher than the industry benchmark of 5%-10% for a massive open online course (MOOC), the findings from this OpenWHO course correspond to other online training experiences for OpenWHO and other virtual training platforms [3-9].

Health care professionals are OpenWHO’s largest user group, accounting for nearly one-third of users [10]. In the context of COVID-19 vaccination, primary health workers may serve as “knowledge ambassadors” [11] or “knowledge brokers” [12] and, as such, may have the greatest chance to increase confidence about the vaccine among their patients. For example, it was demonstrated that the acceptability of the COVID-19 vaccine was greater among individuals who thought their health care provider would recommend it [13].

Online and blended learning can provide substantial cost savings by reducing the need for travel, per diems, and other related expenses, as well as rapidly increasing the potential number of people that can be trained [14,15]. As online learning is still relatively new for the training of health workers, the modality has received mixed reviews. Several systematic reviews report that online learning approaches may be at least as effective as traditional learning approaches [11-14], while others show that online learning may make little or no difference in patient outcomes or health professionals’ behaviors, skills, or knowledge [16]. However, included studies have used different study designs to measure the effectiveness of online learning, from cross-sectional approaches with pre- and post-test assessments (ie, testing before and after the learning activity) [17] to longitudinal research, where knowledge retention was assessed up to 6 months or a 1-year follow up was carried out [18], which makes comparing these studies difficult.

Although the health workers course was well received, feedback did include the need for additional vaccine-specific training content, more translated versions of the course, opportunities to ask questions to technical experts, and the ability to participate in peer-to-peer learning. Following the request for more vaccine-specific content, CRD launched the vaccine-specific resources course in all United Nations languages (Arabic, Chinese, English, French, Russian, and Spanish) as well as Portuguese [19]. This course provides short instructional videos for COVID-19 vaccines that received Emergency Use Listing, such as Pfizer-BioNTech, Moderna, AstraZeneca, and Janssen. In addition, this new course provides job aides (ie, resources providing vaccine-specific information) to support stakeholders involved in COVID-19 vaccine deployment. To support the development of additional language versions, CRD worked with WHO Country Offices to provide translated versions of both this OpenWHO course for health workers and the Orientation to National Deployment and Vaccination Planning for COVID-19 vaccines course [20]. In response to participants’ requests for more interaction and peer learning, CRD developed and implemented the COVID-19 Vaccination: Building Global Capacity webinar series, which brought together technical experts and learners for 15 live sessions dedicated to different aspects of COVID-19 vaccination. This webinar series ultimately reached more than 13,000 learners in 181 countries.

Ideally, virtual training could include recorded and live components, allowing for a combination of the flexibility offered by virtual self-paced learning with the opportunity to interact during the live sessions [21-25]. Considering the speed with which learners completed this course, it may be beneficial to provide shorter versions of the content.

In addition, when considering virtual courses, internet connectivity and the potential for system- or IT-related issues are important to consider, in particular at the subnational level in low-resource settings. In our analysis, nearly one-third of survey respondents noted that they had at least some internet connection issues during their learning. Similarly, recent research demonstrated that accessibility of online learning activities may be hampered by the required baseline level of digital literacy, equipment, and internet connection, which might be of particular importance for certain populations, including refugees or people with vision problems and those living in low-resource settings [26-29]. If the WHO, governments, and partners plan to increase the use of online learning, it is critical to also consider the infrastructure necessary to ensure learners can fully participate.

Limitations of this research include that we focused this analysis on the English-speaking course. Additional analyses could be conducted on the other language versions of the course. This research also reflects a snapshot in time, as the survey was conducted in March and April 2021. Follow-up surveys could be conducted a year or two after the launch of the course to understand the evolution of the course experience and to understand how participants used the information they received from the course in their professional and personal lives. An additional limitation of these findings is the potential bias of people more comfortable with online learning having taken the course and completed the survey. Finally, this analysis may include potential self-reporting bias among survey respondents, while the limited number of questions in the pre- and postquizzes precludes robust statistical analysis of the impact of each module. It would be beneficial for future analyses to consider differences in the characteristics and perspectives of participants who complete virtual courses compared with participants who do not complete virtual courses.

Overall, this analysis highlights a strong interest in online learning among participating health professionals. This willingness to participate in virtual training is important for the WHO and partners to consider when developing educational materials for other vaccine introductions. Online learning may serve as a viable alternative to face-to-face training, particularly in an emergency context when physical distancing is recommended. It would be beneficial for future studies to look at how health workers applied the knowledge gained from this training and to consider the cost-effectiveness and/or cost-benefit of online learning for vaccine introduction, particularly during health emergencies.

### Conclusion

The COVID-19 vaccination trainings were developed for OpenWHO due to the global need for rapidly available training, the need for rapid dissemination to a large number of learners, and the travel and operational limitations posed by the pandemic. This article provides an overview of the usability and utility of this global virtual training, as well as insights from the experience.

In summary, this analysis indicates that this course served its intended purpose of supporting participating health workers in preparing for COVID-19 vaccination deployment. Considering this analysis and the increasing desire of learners to have training materials and performance scores rapidly accessible, Ministries of Health and health facilities should consider the potential of training their health professionals using virtual or blended approaches to increase rapid accessibility and exchange of information [23,25].

## Appendix

Multimedia Appendix 1 Survey questions.

Multimedia Appendix 2 Supplementary table.

## Abbreviations

AEFI: adverse events following immunization
CRD: Country Readiness and Delivery
MOOC: massive open online course
WHO: World Health Organization
