# Supplementary material for: Learning From a Massive Open Online COVID-19 Vaccination Training Experience: Survey Study
Source: JMIR Public Health Surveill. 2021 Dec 3;7(12):e33455. doi: 10.2196/33455 (PMC8647976; doi:10.2196/33455)
Supplement: Multimedia Appendix 1 [file publichealth_v7i12e33455_app1.pdf]

Annex 1: Survey Questions

How was your learning experience? We invite you to complete this survey to help us understand your learning experience with the OpenWHO COVID-19 vaccine introduction courses, and how to enhance it.

Please note that survey answers may be used for research purposes and will be treated anonymously as per common research standards. We appreciate your participation and look forward to receiving your feedback.

1. ***What is your gender?***
   a. Woman
   b. Man
   c. Non-binary
   d. Do not wish to disclose

2. ***What is your age group?***
   a. <20 years
   b. 20-39 years
   c. 40-49 years
   d. 50-59 years
   e. 60-69 years
   f. ≥70 years
   g. Do not wish to disclose

3. ***What is your affiliation?***
   a. Healthcare professional (Physician, nurse etc...)
   b. Public health (Epidemiology, Biostatistics etc...)
   c. WHO, consultant, affiliate
   d. UN country team/ UN partner organization
   e. National ministry of health
   f. Student
   g. Volunteer
   h. Medical research
   i. Non-governmental organization (NGO)/ Non-profit organization
   j. Other – not listed above

4. ***How many years of experience do you have in your field?***
   a. < 1 year
   b. 1-3 years
   c. 4-6 years
   d. 7-9 years
   e. 10-20 years
   f. >20 years

5. ***Where do you currently live?***
    a. List of countries

6. **Are you participating in the Project ECHO COVID-19 Vaccination: Building Global Capacity webinar series?**
    a. Yes
    b. No
    c. Sometimes
    d. Haven't heard of it

7. ***What was your MAIN motivation for taking this course?***
    a. Compulsory requirement
    b. Out of private interest, to gain general knowledge about COVID-19 vaccination with no direct use of the trainings
    c. To help me prepare for specific professional responsibilities related to the COVID-19 vaccine as I expect to play a role and need to be ready
    d. To strengthen my CV or qualification for future employment or assignment
    e. To be able to teach others
    f. Other

8. *The goal of this training was to provide general information on the COVID-19 and specific information on storage, handing and administration of the vaccine, recording and monitoring, and communication to ensure safe and efficient COVID-19 vaccine administration.*
    The content covered the learning goal well.
    ----

9. ***The content of the training was well organized and easy to follow***
    a. Fully agree
    b. Somewhat agree
    c. Somewhat disagree
    d. Fully disagree

10. ***I acquired new knowledge and/or skills from this training package***
    a. Fully agree
    b. Somewhat agree
    c. Somewhat disagree
    d. Fully disagree

11. ***I found the pre- and post-quizzes were helpful/useful***
    a. Fully agree
    b. Somewhat agree
    c. Somewhat disagree
    d. Fully disagree

12. ***What resources in the trainings have you used? (Please select all that apply)***

a. Watched the videos
b. Read the transcripts
c. Downloaded the presentations
d. Downloaded the vaccine specific job aids
e. Other

13. ***After this training, I have more CONFIDENCE in performing my professional role relating to COVID-19 vaccination***
    a. Fully agree
    b. Somewhat agree
    c. Fully disagree
    d. Strongly disagree
    e. Not applicable to me

14. ***Were there any barriers to this course learning? (Please select all that apply)***
    a. Internet connection issues (No internet access, unstable internet, paid internet, etc.)
    b. System or IT-related issues (Brower issue, page freeze, OpenWHO down, etc.)
    c. Difficulty navigating pages (finding information quickly)
    d. Not enough time to complete the course
    e. Language barrier (not offered my own language)
    f. No barriers
    g. Others

15. ***After this experience with OpenWHO online learning, I prefer***
    a. Online training courses
    b. Classroom (in-person) training courses
    c. Combination of online and classroom (in-person) training courses
    d. I don't know

16. ***Please tell us why you prefer the method of training you selected above (Select all that apply)***
    a. I can take the course at any convenient time
    b. I can take the course at my own pace
    c. I can replay sections of the course
    d. I can download the materials from the course
    e. I prefer courses with face-to-face interaction
    f. I prefer a group learning environment where several employees learn at the same time
    g. It provides greater ability to concentrate
    h. Other reasons

17. ***Would you recommend this training course to others?***
    a. Fully agree
    b. Somewhat agree

c. Somewhat disagree
d. Fully disagree

18. I am satisfied with the overall training contents of this training package

a. Fully agree
b. Somewhat agree
c. Somewhat disagree
d. Fully disagree

19. What area do we need to improve the most?

a. This course should be offered in more languages
b. OpenWHO should offer more courses on COVID-19 vaccination
c. This course does not apply to my local context
d. The course website is difficult to use or navigate
e. This course is not interactive enough or does not use enough animation
f. This course is too easy
g. This course is too difficult
h. The assessments are too easy
i. The assessments are too difficult
j. Other
k. No improvements needed

20. Are there topics that you would have liked to see addressed that were not covered in this training module?

[Please type here]

21. WHO plans to conduct in-depth online interviews with some course participants. Are you willing to be contacted by WHO to share your learning experience in this course?

a. YES, I agree that I may be contacted by WHO to share my learning experience
b. No, I do not wish to be contacted by WHO about my learning experience
