# Supplementary material for: Learning From a Massive Open Online COVID-19 Vaccination Training Experience: Survey Study
Source: JMIR Public Health Surveill. 2021 Dec 3;7(12):e33455. doi: 10.2196/33455 (PMC8647976; doi:10.2196/33455)
Supplement: Multimedia Appendix 2 [file publichealth_v7i12e33455_app2.pdf]

Multimedia Appendix 2. Supplementary table.

Table S1. Motivation for taking the health workers course by selected countries, affiliations, and years of experience from survey participants data (total survey participants = 2,019; survey period from 19 March to 09 April 2021)*

	To help me prepare for specific professional responsibilities	Compulsory requirement	To be able to teach others	Out of private interest	To strengthen my CV	Other
By country						
Philippines (n=268)	186 (69.7%)	38 (14.2%)	5 (1.9%)	18 (6.7%)	17 (6.4%)	3 (1.1%)
USA (n=190)	89 (47.9%)	59 (31.7%)	1 (0.5%)	12 (6.5%)	14 (7.5%)	11 (5.9%)
Botswana (n=123)	94 (77.1%)	5 (4.1%)	9 (7.4%)	6 (4.9%)	7 (5.7%)	1 (0.8%)
By affiliation						
Healthcare professional (n=1,348)	845 (63.4%)	134 (10.1%)	78 (5.9%)	163 (12.2%)	96 (7.2%)	16 (1.2%)
Students (n=224)	97 (43.9%)	55 (24.9%)	11 (5.0%)	21 (9.5%)	25 (11.3%)	12 (5.4%)
National ministry of health (n=42)	26 (61.9%)	0 (0%)	9 (21.4%)	2 (4.8%)	5 (11.9%)	0 (0%)
By years of experience						
<1 year (n=254)	127 (50.6%)	41 (16.3%)	17 (6.8%)	28 (11.2%)	32 (12.7%)	6 (2.4%)
4-6 years (n=306)	190 (62.3%)	32 (10.5%)	16 (5.2%)	36 (11.8%)	26 (8.5%)	5 (1.7%)
>20 years (n=311)	189 (62.2%)	26 (8.6%)	28 (9.2%)	42 (13.8%)	15 (4.9%)	4 (1.3%)

*Percentages are based on total number of non-missing responses
